# IL-17RA Signaling Amplifies Antibody-Induced Arthritis

**DOI:** 10.1371/journal.pone.0026342

**Published:** 2011-10-20

**Authors:** Christian D. Sadik, Nancy D. Kim, Elena Alekseeva, Andrew D. Luster

**Affiliations:** Center for Immunology and Inflammatory Diseases, Division of Rheumatology, Allergy, and Immunology, Massachusetts General Hospital, Harvard Medical School, Boston, Massachusetts, United States of America; Oklahoma Medical Research Foundation, United States of America

## Abstract

**Objective:**

To investigate the role of IL-17RA signaling in the effector phase of inflammatory arthritis using the K/BxN serum-transfer model.

**Methods:**

Wild-type and *Il17ra^−/−^* mice were injected with serum isolated from arthritic K/BxN mice and their clinical score was recorded daily. Mice were also harvested on days 12 and 21 and ankles were analyzed for cytokine and chemokine mRNA expression by qPCR on day 12 and for bone and cartilage erosions by histology on day 21, respectively. The induction of cytokine and chemokine expression levels by IL-17A in synovial-like fibroblasts was also analyzed using qPCR.

**Results:**

*Il17ra^−/−^* mice were partially protected from clinical signs of arthritis and had markedly fewer cartilage and bone erosions. The expression of several pro-inflammatory mediators, including the chemokines KC/CXCL1, MIP-2/CXCL2, LIX/CXCL5 MIP-1γ/CCL9, MCP-3/CCL7, MIP-3α/CCL20, the cytokines IL-1β, IL-6, RANKL and the matrix metalloproteinases MMP2, MMP3, and MMP13 were decreased in the ankles of *Il17ra^−/−^* mice compared to wild-type mice. Many of these proinflammatory genes attenuated in the ankles of *Il17ra^−/−^* mice were shown to be directly induced by IL-17A in synovial fibroblasts *in vitro*.

**Conclusions:**

IL-17RA signaling plays a role as an amplifier of the effector phase of inflammatory arthritis. This effect is likely mediated by direct activation of synovial fibroblasts by IL-17RA to produce multiple inflammatory mediators, including chemokines active on neutrophils. Therefore, interrupting IL-17RA signaling maybe a promising pharmacological target for the treatment of inflammatory arthritis.

## Introduction

The IL-17 cytokine family has been implicated in the pathogenesis of numerous autoimmune diseases, including rheumatoid arthritis [1]. IL-17A and IL-17F are the most homologous (55%) members of this family and exhibit overlapping functions [2]. Both cytokines bind to the same IL-17 receptor complex consisting of the receptor subunits IL-17RA and IL-17RC [3], [4]. IL-17A and IL-17F are both expressed in the joints of RA patients [5]–[10]. Likewise, the expression of their receptor subunits, IL-17RA and IL-17RC, is enhanced in the blood and synovium of RA patients [11]. Therefore, IL-17A and IL-17F may both participate in the pathogenesis of rheumatoid arthritis.

IL-17A has been implicated in arthritogenesis in several mouse models of RA [12]–[16]. Notably, the relative importance of IL-17A in these models differs: the more T cell-dependent the model, the more pivotal the role of IL-17RA signaling [17]. This suggests that IL-17A is particularly involved in the initiation phase of arthritis, generally characterized by the generation of an autoimmune response. For example, in the spontaneous K/BxN mouse model, IL-17A was required for the generation of anti-GPI autoantibodies [18].

In the K/BxN serum transfer model of inflammatory arthritis, the initiation phase of arthritis is bypassed by passive transfer of anti-GPI-containing K/BxN serum into recipient mice, which induces acute arthritis. Thus, the K/BxN serum transfer model allows studies to focus specifically on the effector phase of inflammatory arthritis, which is primarily driven by innate immune cells [19], [20]. While T cells are generally not required for the induction of acute arthritis in this serum transfer model, it has recently been shown that arthritis in B cell-deficient BxN mice is reinforced by adoptive transfer of KRN T cells [21]. This reinforcement depended on the release of IL-17A [21]. The molecular mechanism of this reinforcement, however, was not addressed, nor was the involvement of IL-17RA signaling in the K/BxN serum transfer model.

In human RA, the synovial fluid of inflamed joints is largely infiltrated by neutrophils. Likewise, neutrophils dominate the synovial fluid infiltrate in inflamed joints in both the spontaneous K/BxN model and the K/BxN serum transfer model, and neutrophils have been shown to be indispensable for the generation of serum-induced arthritis [20], [22]. IL-17A and IL-17F have been shown to promote the infiltration of neutrophils into inflammatory sites [23] and to induce the expression of neutrophil-active chemokines from stromal cells, such as human fibroblast-like synoviocytes (FLS) [9], [11].

In this study, we addressed the role of IL-17RA signaling in the effector phase of arthritis using the K/BxN serum transfer model and show that IL-17RA signaling reinforces destructive arthritis. We found that IL-17RA contributes to the effector phase of arthritis through the direct induction of neutrophil-active chemokines, RANKL, and the matrix metalloprotease MMP3 in FLS.

## Materials and Methods

### Animals

Previously described *Il17ra^−/−^* mice on the C57BL/6 background [23] were kindly provided by Amgen (Seattle, WA) and bred under specific pathogen free conditions, which included Helicobacter pylori and Pasteurella pneumotropica (HPP), at the Massachusetts General Hospital. HPP-free wild-type *C57BL/6* mice were purchased from The Jackson Laboratory (Bar Harbor, ME). KRN mice were kindly provided by Diane Mathis and Christophe Benoist (Harvard Medical School, Boston, MA). K/BxN mice were obtained by crossing KRN with NOD/LtJ mice (The Jackson Laboratory, Bar Harbor, ME) in our animal facility. All experiments were performed according to protocols approved by the Massachusetts General Hospital Subcommittee on Research Animal Care. Age- and sex-matched, 6–12 week old mice were used in all experiments.

### Serum transfer and clinical evaluation

K/BxN serum was harvested from 8-week-old arthritic K/BxN mice, pooled and stored at −80°C until usage. For induction of arthritis 150 µl of serum was injected i.p. into recipient mice on days 0 and 2 of the experiment. The clinical score for each paw was evaluated at least every second day based on the following index: 0, no edema/erythema; 1, localized edema/erythema over one surface of the paw; 2, edema/erythema involving the entirety of one surface of the paw; 3, edema/erythema involving both surfaces of the paw. Scores were added for all four paws to obtain a composite score with a maximum of 12. Ankle thickness was determined with a pocket thickness gage (Mitutoyo USA, Aurora, IL) and ankle thickening (ankle swelling compared to baseline on day 0) was calculated as the mean difference between the current ankle thickness and the ankle thickness of each hindpaw on day 0 before serum injection.

### Histopathology

Mice were sacrificed on day 7 and day 21. Ankles were dissected and fixed in 4% neutral buffered paraformaldehyde, demineralized in modified Kristensen's solution, and stained with toluidine blue. Inflammation, cartilage and bone erosions were scored as described with 0, normal; 1, minimal; 2, mild; 3, moderate; 4, marked; 5, severe [24].

### Determination of the number of neutrophils in the synovial fluid

Ankles were dissected on day 12 after serum transfer and lavaged. The infiltrates from both ankles of an individual mouse were combined and stained with anti-Ly6G FITC (R&D Systems, Minneapolis, MN). Afterwards, counting beads (Invitrogen, Carlsbad, CA) were added according to manufacturer's instructions and the number of Ly-6G^+^ cells was determined by FACS analysis.

### RNA isolation and qPCR

Total RNA was isolated using TRIzol (Invitrogen, Carlsbad, CA) and treated with DNase I (Invitrogen, Carlsbad, CA) according to the manufacturers' instructions. The total RNA concentration was determined with a Nanodrop (ThermoFisher Scientific, Waltham, MA) and total RNA was reverse transcribed using oligo(dT), random hexamers, and multiscribe reverse transcriptase (Applied Biosystems, Foster City, CA). QPCR was performed using 1 µl cDNA per well, SYBR green master mix (Applied Biosystems, Foster City, CA), and sense and antisense primers 250 nmol each. All primers for qPCR were purchased from Integrated DNA Technologies (Coralville, IA) and primer sequences are listed in Table S1. QPCR was conducted with the MX4000 qPCR machine (Stratagene). Data were analyzed using MX4000 software version 3.0 (Stratagene). Results were evaluated using the ΔΔC_T_ method and the calculated number of copies was normalized to the number of β_2_ microglobulin mRNA copies in the same sample.

### Comparison of mRNA expression in the ankle joints of wild-type and *Il17ra^−/−^* mice after serum-transfer

Arthritis was induced in WT and *Il17ra^−/−^*, as described above. On day 12, for the generation of whole joint lysates, joints were dissected, immediately put into TRIzol and minced using a polytron at 4°C. Total RNA was isolated and DNase I treated, as described above. For reverse transcription, 1 µg of total RNA was used.

### Isolation of murine bone marrow-derived neutrophils and synovial-like fibroblasts (FLS)

Bone marrow-derived neutrophils used for *in vitro* experiments were isolated using an immunomagnetic separation strategy. Freshly harvested mouse bone marrow leukocytes were first stained with PE-conjugated anti-Ly6G (BD Biosciences, San Jose, CA) and then isolated using EasySep® PE selection kits (Stem Cell Technologies, Vancouver, Canada) and immediately used for experiments. Synovial-like fibroblasts (FLS) were obtained from C57Bl/6 mouse ankle tissues. Dissected ankle tissues were infiltrated and digested in Type IV collagenase (Worthington Corporation, Lakewood, NJ). After an overnight culture in tissue culture flasks, non-adherent cells were washed away and adherent cells were maintained in DMEM supplemented with 10% heat-inactivated FCS, 2 mM glutamine, 100 U/ml penicillin, 100 µg/ml streptomycin, and 50 µM 2-mercaptoethanol. Fibroblast monolayers were cultured until confluent and used between the fourth and eighth passages.

### Stimulation of FLS with IL-17A

150,000 FLS per well were seeded into 12-well plates (Corning, Lowell, MA). After 2 days, cells were washed and 1.5 ml fresh medium was added. Cells were either left untreated or stimulated with 100 ng/ml IL-17A (R&D Systems, Minneapolis, MN). After 16 h, total RNA was harvested and 0.5 µg RNA were used for reverse transcription.

### Chemotaxis assays

Chemotaxis of neutrophils was assessed *in vitro* using both 24-well transwell plates (Corning; Lowell, MA) and the ChemoTx® System (Neuro Probe, Gaithersburg, MD). Freshly prepared bone marrow neutrophils were suspended in RPMI containing 2 nM glutamine, 25 nM HEPES and 1% fatty-acid free BSA at a concentration of 5×10^6^ cells/ml and chemotaxis was assessed to varying concentrations of IL-17A (R&D Systems, Minneapolis, MN). 100 nM leukotriene B_4_ (Cayman Chemical, Ann Arbor, MI) or 100 nM recombinant murine MIP-2 (R&D Systems) served as positive controls. Chemotaxis was quantified after incubation for 1 h at 37°C, 5% CO_2_.

For assays using 24-well transwell plates, 100 µl of the neutrophil suspension was seeded into the insert of a 24-well co-culture plate with a pore size of 5 µm. Migrated cells were harvested from the lower well, spun down and resuspended in PBS. Counting beads (Invitrogen, Carlsbad, CA) were added according to manufacturer's instructions and the absolute number of neutrophils in each sample was assessed by FACS analysis. For assays using the ChemoTx® System, chemoattractants were placed in the bottom chamber of a 96-well ChemoTx disposable plate and neutrophils were seeded on the top of a 3 µm pore size membrane. For chemokinesis controls, chemoattractants were added to the cells on top instead of into the bottom chamber. Neutrophils that migrated into the lower chamber after 1 h of incubation at 37°C, 5% CO_2_ were counted with an inverted microscope and the chemotactic index (CI) was calculated by establishing the ratio between the number of cells that migrated in the presence of a chemoattractant to the number of cells that migrated in complete absence of a chemoattractant (medium control).

### Statistics

All data are presented as mean ± SEM. Raw data were analyzed by unpaired two-tailed Student's *t* test or Mann-Whitney-Wilcoxon test, as indicated. In case of more than two groups, data were analyzed by One-way-ANOVA with Bonferroni posttest. A value of *p*<0.05 was considered statistically significant. To test for statistical differences between the wild-type and *Il17ra^−/−^* mice in ankle thickness and clinical score, the area under curve (AUC) for every individual mouse in each group was calculated and their means were compared. All calculations were performed with GraphPad Prism 4.0 software.

## Results

### Serum-induced arthritis is attenuated in *Il17ra^−/−^* mice

**Figure 1:**
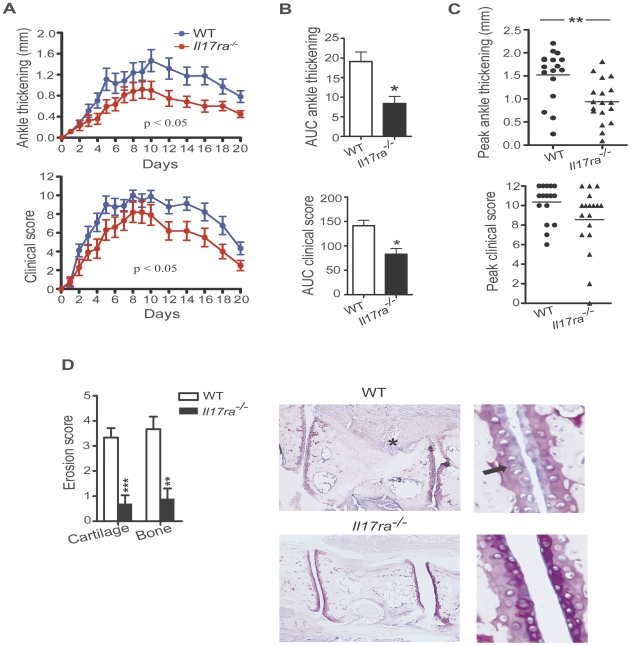
IL-17RA deficiency attenuates severity of K/BxN serum induced arthritis. **A,** WT (n = 9) and *Il17ra^−/−^* mice (n = 10) were injected with K/BxN serum and were monitored for 20 days recording daily ankle thickness and clinical score. Data are presented as mean ± SEM. The AUC for the ankle thickness and for the clinical score of every individual mouse was calculated and statistical differences between wild-type and *Il17ra^−/−^* mice were determined by unpaired two-tailed Student's *t* test. One representative of three independent experiments is shown. The number of mice stated above refers to this one representative experiment. **B,** AUCs for the ankle thickness and the clinical score of WT (n = 17) and *Il17ra^−/−^* mice (n = 18) were calculated. Each column represents mean ± SEM. Data were pooled from three independent experiments, and statistical differences were determined by unpaired two-tailed Student's *t* test. **C,** Peak ankle thickness and the peak clinical score reached by each WT (•) (n = 17) and *Il17ra^−/−^* (▴) mice (n = 18) are shown. Each dot or triangle represents one individual mouse. Data were pooled from three independently performed experiments. **D,** Histopathological score of cartilage and bone erosions in WT (n = 9) and *Il17ra^−/−^* mice (n = 10) are presented as mean ± SEM from ankles harvested on day 21. Data shown are from one representative out of three independently performed experiments. Histopathological score was assessed in toluidine-blue stained specimens. Representative histologies for the wild-type and *Il17ra^−/−^* groups are shown. The asterisk marks a site of pannus infiltration into the bone already visible under low magnification. The arrow indicates a site of proteoglycan loss of the cartilage. Both findings were typical for the wild-type group but not for the *Il17ra^−/−^* group. In (**C**) and (**D**) statistical analyses were conducted using Mann-Whitney-Wilcoxon test. * = p<0.05; ** = p<0.01; *** = p<0.001.

To determine the role of IL-17RA signaling in the effector phase of inflammatory arthritis, we analyzed the development of arthritis in *Il17ra^−/−^* mice compared to wild-type mice following the transfer of arthritogenic K/BxN serum transfer. Clinical signs of arthritis developed in both groups of mice with a similar time course. However, the clinical signs of arthritis were attenuated in *Il17ra^−/−^* mice compared to wild-type mice throughout the entire course of the disease (Fig. 1A). The difference was more pronounced in ankle thickness than in the clinical score. *Il17ra^−/−^* mice showed approximately 40% less ankle thickness than wild-type mice at the peak of ankle thickening on day 10 (Fig. 1A). Calculating the area under the curve (AUC) for both ankle thickness and the clinical score for every individual mouse and comparing the means of the WT and the *Il17ra^−/−^* groups showed a statistically significant difference for both parameters. The comparison of the AUCs of three independent experiments was different in both clinical scoring and ankle thickness with a reduction of approximately 42% and 58% for the clinical score and the ankle thickening, respectively (Fig. 1B). Comparison of the peak clinical score and the peak ankle thickness of the individual mice of the same three experiments showed a decrease in peak ankle thickness in *Il17ra^−/−^* mice as well (Fig. 1C). There was a trend towards a lower peak clinical score in *Il17ra^−/−^* mice, although it did not reach statistical significance. Histopathological scoring for cartilage and bone erosions in wild-type and *Il17ra^−/−^* mice 21 days after serum transfer revealed that *Il17ra^−/−^* mice had markedly reduced cartilage and bone erosions compared to wild-type mice (Fig. 1D). Histological evidence of inflammation was not different between these groups of mice and by 21 day evidence of inflammatory cell infiltration had almost completely resolved in both groups (data not shown).

### Reduced numbers of neutrophils in the joints of *Il17ra^−/−^* mice

**Figure 2:**
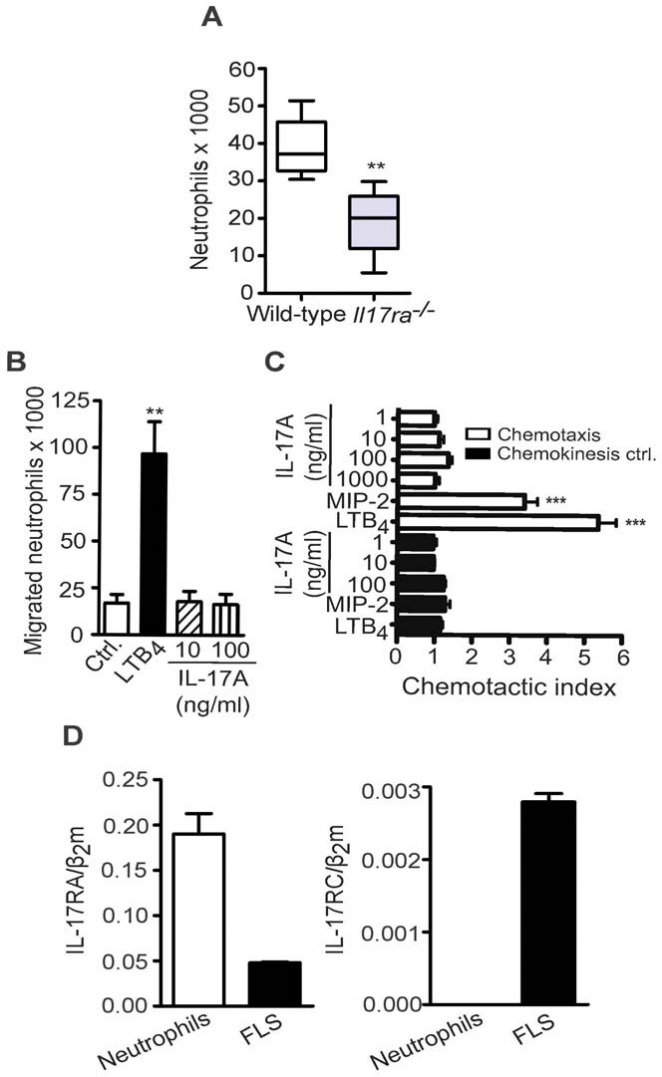
Neutrophils are reduced in the joints of *Il17ra^−/−^* mice and are unresponsive to direct stimulation with IL-17A. **A,** Number of neutrophils in the ankle joints of WT and *Il17ra^−/−^* mice on day 12 was determined by FACS analysis counting Ly6G^+^ cells in relation to counting beads. Data are presented as mean ± SEM (n = 3 mice per group). One of three independent experiments is shown. **B,** Chemotaxis of freshly isolated murine bone marrow-derived neutrophils towards 100 nM LTB_4_ and IL-17A (10 and 100 ng/ml) assessed using 24-well transwell assays. Data represent numbers of migrated neutrophils (n = 3 independently performed experiments). **C,** Chemotaxis of freshly isolated murine bone marrow-derived neutrophils towards LTB_4_ (100 nM) MIP-2 (100 nM) and IL-17A (1, 10, 100, 1000 ng/ml) as well as their corresponding chemokinesis controls assessed using 96-well ChemoTx assays. Data are presented as chemotactic index (number of cells migrating to chemoattractant/number of cell migrating to medium control). Data shown are mean ± SEM (n = 4 independently performed experiments). **D,** Levels of IL-17RA and IL-17RC mRNA determined by qPCR on RNA isolated from murine FLS and freshly isolated bone marrow-derived neutrophils (n = 3 independently performed experiments). Data were compared by unpaired two-tailed Student's *t* test, ** = p<0.01 compared to wild-type control (**A**), or by One-way-ANOVA with Bonferroni posttest, ** = p<0.01 compared to medium control (**B**) or *** = p<0.001 LTB_4_ and MIP-2 each individually compared to all other groups (**C**).

Since the synovial infiltrate in serum-induced arthritis is largely neutrophils, we determined the number of neutrophils found in the synovial fluid on day 12 after serum transfer. Day 12 was chosen because the difference in arthritis between the WT and the *Il17ra^−/−^* group was most pronounced at this time point. To determine the number of neutrophils present in the synovial fluid of the ankle, leukocytes recovered from joints by lavage were stained for Ly6G^+^ cells to identify neutrophils. The number of neutrophils in the sample was determined using FACS analysis in the presence of counting beads. The synovial fluid of *Il17ra^−/−^* mice was infiltrated by fewer neutrophils than the synovial fluid of wild-type mice (Fig. 2A).

There are conflicting data in the literature concerning the direct responsiveness of neutrophils to IL-17A [25], [26]. Therefore, we investigated the direct effect of IL-17A on neutrophils. Stimulation of neutrophils with IL-17A (100 ng/ml) for 3 h, 16 h or 24 h had no effect on gene expression of IL-6, numerous chemokines, RANKL, MMP2, MMP3, MMP13, IL-1β or TNF-α (data not shown). We also tested if varying concentrations of IL-17A could induce migration of freshly isolated murine bone marrow neutrophils in chemotaxis assays (Fig. 2B). While LTB_4_ and MIP-2 induced significant neutrophil migration and served as positive controls, IL-17A failed to induce neutrophil chemotaxis. Therefore, it is unlikely that IL-17A directly induces significant neutrophil recruitment *in vivo*. This assumption was further supported by the fact that murine neutrophils do not express IL-17RC mRNA, although they do express IL-17RA mRNA (Fig. 2C). Combined expression of IL-17RA and IL-17RC is a prerequisite for a functional IL-17 receptor complex in both mice and humans [26]–[28].

### The induction of pro-inflammatory mediators in the joint is attenuated in *Il17ra^−/−^* mice

On day 12 following arthritogenic serum transfer, joints of wild-type and *Il17ra^−/−^* mice were harvested and total RNA, isolated from whole ankle joint lysates, was used for gene expression analysis by qPCR. Compared to wild-type mice, several genes were markedly attenuated in the ankles of *Il17ra^−/−^* mice (Table 1). The expression of the pro-inflammatory cytokines IL-1β and IL-6 was significantly lower. IL-1β expression in the joint has previously been shown to be essential for the development of serum-induced arthritis with neutrophils as a major source for this cytokine [29], [30]. We have recently shown that CCR1 and CXCR2 are needed for the development of full-blown serum-induced arthritis. Consistent with these previous data, *Il17ra^−/−^* mice had diminished expression of the CXCR2 ligands KC/CXCL1, MIP-2/CXCL2, and LIX/CXCL5, and the CCR1 ligands MIP-1α/CCL3, MIP-1γ/CCL9, and MCP-3/CCL7 (Table 1).

**Table 1:** Gene expression in the ankles of WT and IL-17RA^−/−^ mice.

	mRNA copies/β2 microglobulin×100		
	WT	IL-17RA^−/−^	p-value
**IL-1β**	**4.7±0.67**	**1.2±0.39**	**0.0001**
IL-6	2.5±1.2	0.2±0.08	0.0599
**KC/CXCL1**	**2.8±0.8**	**0.5±0.3**	**0.0136**
**MIP-2/CXCL2**	**5.5±1.1**	**1.7±0.7**	**0.0104**
**LIX/CXCL5**	**9.2±1.6**	**2.6±1.0**	**0.0017**
**MIP-1γ/CCL9**	**1.5±0.3**	**0.7±0.3**	**0.0213**
**MIP-3α/CCL20**	**0.2±0.04**	**0.06±0.02**	**0.0018**
MIP-1α/CCL3	0.5±0. 1	0.2±0.06	0.0533
RANTES/CCL5	3.4±1.2	4.5±2.1	0.6589
**MCP-3/CCL7**	**9.0±1.7**	**2.3±0.7**	**0.0014**
MIP-3β/CCL19	1.0±0.2	0.9±0.2	0.6408
**RANKL**	**0.8±0.2**	**0.3±0.1**	**0.0435**
IL-33	1.9±0.6	0.9±0.6	0.1343
VEGF-A	1.8±0.4	1.0±0.2	0.0536
**MMP2**	**45.1±9.4**	**18.2±7.8**	**0.0379**
**MMP3**	**22.5±4.7**	**7.1±2.9**	**0.0106**
MMP13	11.6±3.1	4.7±1.5	0.0562

RNA expression in the ankles of wild-type and *Il17ra^−/−^* mice after serum-transfer. WT and *Il17ra^−/−^* mice were injected with K/BxN serum. On day 12, total RNA was harvested from whole ankle lysates and RNA levels assessed by qPCR. Data were normalized to the number of β_2_ microglobulin mRNA copies. Results are presented as the 100-fold of the mean of mRNA copies/β_2_ microglobulin mRNA copies ± SEM (n = 13 mice per group). Data are compiled from three independent experiments. Differences were evaluated by unpaired two-tailed Student's *t* test; p<0.05 was considered statistically significant and is indicated in bold.

MIP-3α/CCL20 is the only known chemokine ligand for CCR6, a chemokine receptor expressed on Th17 and γδ T cells. Both cell types are potential sources for IL-17A and IL-17F. We found MIP-3α/CCL20 mRNA expression was enhanced in the inflamed joints and that IL-17RA deficiency blunted the expression of this chemokine in the joint (Table 1). Recently, IL-33 has been linked to arthritis and the recruitment of neutrophils into the joint. Synovial cells are considered as the major source for IL-33 in arthritis [31]–[33]. Significant levels of IL-33 mRNA were detected in inflamed joints after injection of K/BxN serum. Those levels tended to be decreased in the joints of *Il17ra^−/−^* mice compared to wild-type mice (Table 1), suggesting a possible link between those two amplifiers of arthritis. Expression of IL-17A has also been linked to bone and cartilage erosions through the induction of RANKL, which is induced in inflamed joints following serum transfer [24]. We found that RANKL expression was inhibited in the absence of IL-17RA signaling (Table 1). Likewise, *Il17ra^−/−^* mice had reduced mRNA levels of the matrix metalloproteinases MMP2, MMP3, and MMP13 (Table 1). There was also a trend towards lower VEGF-A levels in the joints of *Il17ra^−/−^* mice following serum transfer (Table 1). VEGF-A is a critical mediator of angiogenesis and has previously been linked to arthritis development K/BxN mice [34].

### IL-17A induces neutrophil-active chemokines and MMPs in FLS *in vitro*

Synovial cells play a crucial active role in the development of RA, and IL-17A has previously been shown to directly activate synovial cells *in vitro* [11], [13]. In order to determine which of the genes downregulated in the ankles of *Il17ra^−/−^* mice might be directly regulated by IL-17RA signaling, murine fibroblast-like synoviocytes (FLS) were either left unstimulated or stimulated with 100 ng/ml IL-17A for 16 h. RNA was then isolated and gene expression assessed by qPCR. IL-17RA signaling in FLS induced a number of genes, which were down-regulated in the joints of *Il17ra^−/−^* mice, suggesting that IL-17A directly regulated these genes *in vivo*. In particular, the expression of the neutrophil-active CXCR2 chemokines ligands KC/CXCL1 and LIX/CXCL5 was strongly induced by IL-17A in FLS. The neutrophil active CCR1 ligand CCL9/MIP-1γ was also induced but to a lesser extent. In addition, the expression of IL-6, MCP-3/CCL7 and the CCR6 ligand MIP-3α/CCL20 and was also induced by IL-17A in FLS (Table 2). Of note, the induction of the CCR6 ligand CCL20 by IL-17A in FLS may serve as a positive feedback loop to reinforce IL-17-driven inflammation by recruiting IL-17 producing CCR6-expressing Th17 and γδT cells. MIP-3α/CCL20 can be detected in the synovium of RA patients [35], however, a possible role of MIP-3α/CCL20 in serum-induced arthritis has never been addressed.

**Table 2:** Gene expression in FLS after stimulation with IL-17A.

	mRNA copies/β2 microglobulin×100		
	Control	IL-17A	p-value
IL-1β	n.d.	n.d.	-
**IL-6**	**0.065±0.01**	**2.1±0.3**	**0.003**
**KC/CXCL1**	**6.4±0.6**	**80.4±18.1**	**0.015**
MIP-2/CXCL2	0.14±0.03	0.33±0.09	0.102
**LIX/CXCL5**	**27.8±1.7**	**641.9±123.5**	**0.008**
**MIP-1γ/CCL9**	**1.1±0.1**	**2.0±0.2**	**0.015**
**MIP-3α/CCL20**	**0.02±0.004**	**0.74±0.16**	**0.011**
MIP-1α/CCL3	0.17±0.02	0.14±0.03	0.360
MIP-3β/CCL19	0.44±0.05	0. 80±0.13	0.059
**MCP-3/CCL7**	**40.7±4.4**	**252.4±49.5**	**0.013**
RANTES/CCL5	0. 32±0.086	0.60±0.09	0.091
**RANKL**	**1.37±0.05**	**7.4±1.1**	**0.006**
IL-33	1.9±0.14	3.15±0.48	0.068
VEGF-A	20.5±1.4	24.0±5.3	0.553
MMP2	277.3±38.4	271.6±41.4	0.924
**MMP3**	**115.3±16.9**	**339.5±45.0**	**0.010**
MMP13	103.3±3.3	249.2±71.3	0.110

Genes induced in FLS by IL-17A. FLS were left unstimulated or stimulated with 100 ng/ml IL-17A. After 16 h, total RNA was harvested and gene expression was determined by qPCR. Data shown represent the 100-fold of the mean of mRNA copies/β_2_ microglobulin mRNA copies ± SEM (n = 3 independent experiments). Differences were evaluated by unpaired two-tailed Student's *t* test; p<0.05 was considered statistically significant and is indicated in bold.

Importantly, IL-17A also markedly induced the expression of genes linked to cartilage and bone erosions, such as RANKL and the matrix metalloproteinases MMP3 (Table 2) [24]. Stimulation with IL-17A did not affect the expression of VEGF-A, MIP-1α/CCL3, or MMP2, while MIP-2/CXCL2, IL-33 and MMP13 appeared to be minimally induced (∼2-fold) (Table 2). IL-1β mRNA expression was undetectable in FLS.

## Discussion

In this study, we addressed the contribution of IL-17RA signaling to the effector phase of arthritis in the K/BxN serum transfer model by subjecting *Il17ra^−/−^* mice to serum transfer and found that IL-17RA deficiency attenuated the severity of serum-induced arthritis. Clinical signs of arthritis, cartilage and bone erosions, and the expression of chemokines and MMPs in the joints were all reduced. Thus, IL-17RA signaling appears to be involved in the effector phase of serum-induced arthritis. IL-17RA deficiency, however, did not completely abrogate arthritis. Instead, the presence of other pro-inflammatory cytokines, such as IL-1β, was sufficient to generate arthritis, leaving IL-17RA signaling as an amplifier of serum-induced arthritis rather than an absolutely required mediator. The notion that IL-17RA amplifies of arthritis is further supported by the observation that overexpression of IL-17A aggravates serum-induced arthritis [36].

In *Il17ra^−/−^* mice, the biological activity of both IL-17A and IL-17F is completely abrogated, so residual disease in *Il17ra^−/−^* mice is mediated by cytokines other than IL-17A or IL17F. The overlapping roles of IL-17A and IL-17F may explain why Jacobs et al. [21] did not detect a decrease in ankle thickening in serum-induced arthritis in *C57BL/6* mice treated with an antibody to just IL-17A. Another explanation for these discrepant results may be that we observed that the attenuation of arthritis in *Il17ra^−/−^* mice was most pronounced from day 10 through day 20, after arthritis had already reached its peak in both groups. Jacobs *et al.* injected anti-IL-17A antibody on days 0, 2, and 4. Conceivably, on days 10 to 20, the anti-IL-17A antibody concentration was already too low to inhibit IL-17A sufficiently.

Although Th17 cells are the most prominent source of IL-17A and IL-17F, they are not the only source for the two cytokines. Instead, a broad array of cells of the innate and adaptive immune system, including γδ T cells, CD8^+^ T cells, natural killer (NK) and natural killer T (NKT) cells, monocytes and neutrophils, are capable of producing these two cytokines [28], [37]. For instance, in collagen-induced arthritis, γδ T cells have been shown to be the major source of IL-17A in the inflamed joint [38]. The induction of IL-17A in γδ T cells was not dependent on engagement of the T cell receptor but on activation of the γδ T cells by IL-1β and IL-23 [38]. Most intriguingly, a recent study identified tissue mast cells as the major source for IL-17A in RA patients [39]. The release of IL-17A from mast cells could be induced by stimulation with immune complexes or C5a [39]. Since mast cells are believed to participate in the induction of serum-induced arthritis *via* activation of their Fcγ receptors and the C5aR [40], [41], it is tempting to speculate that they are also an important source of IL-17 family cytokines in this mouse model.

We have recently shown that CCR1 and CXCR2 play crucial non-redundant roles in serum-induced arthritis and together account for all of the neutrophil chemokine activity in the model [30]. While deficiency in CCR1 delayed the initiation of arthritis and slightly attenuated its maximal expression, *Cxcr2^−/−^* mice exhibited a nearly indistinguishable early phase of arthritis compared to wild-type mice but in later stages showed a marked attenuation of neutrophil recruitment into the joint and disease. In the joints of *Il17ra^−/−^* mice, mRNA levels of CCR1 and CXCR2 ligands were markedly diminished with attenuation of CXCR2 ligands being more pronounced. Accordingly, although arthritis in *Il17ra^−/−^* mice was attenuated in all phases, the more pronounced clinical difference was found in the second phase of arthritis when the arthritis is amplified and maintained by CXCR2 chemokine ligands. In addition, IL-17A potently induced these CXCR2 chemokine ligand mRNA in FLS *in vitro*, suggesting a direct mechanism of neutrophil active chemokine induction by IL-17A in synovial cells *in vivo*. Their lower expression in the inflamed joints of *Il17ra^−/−^* mice therefore could explain the diminished number of neutrophils found in the synovial fluid of *Il17ra^−/−^* mice compared to wild-type mice. It should be noted that chemokine mRNA levels and not proteins levels were analyzed so caution should been used when interpreting these data. Nevertheless, these data suggest that the local induction of CXCR2 and CCR1 chemokines in the synovium is likely the major mechanism of the amplification of serum-induced arthritis by IL-17RA signaling. Consistent with this, it has been shown that the acute infiltration of the joint by neutrophils after an intraarticular re-challenge with methylated bovine serum albumin (mBSA) depends on the local production of IL-17A and the subsequent downstream release of LTB_4_, CXCL1, CXCL5, and TNF-α [27]. In this study, however, a direct effect of IL-17A on neutrophils was proposed with IL-17A exerting a chemotactic effect on neutrophils *in vitr*o dependent on the autocrine release of CXCL1. In our experiments, neutrophils were not responsive to direct stimulation with IL-17A. IL-17A did not induce neutrophil chemotaxis or alter gene expression profiles *in vitro*. Recent literature on the direct responsiveness of neutrophils to IL-17A is conflicting. Other investigators have reported that IL-17A is unable to directly stimulate human neutrophils [26]. Deficiency of IL-17RA signaling resulted in a marked reduction in cartilage and bone erosions. The development of cartilage and bone erosions in serum-induced arthritis is critically dependent on RANKL [24]. Consistent with this, RANKL mRNA expression was strongly reduced in the joints of *Il17ra^−/−^* mice. Furthermore, IL-17A directly induced RANKL in FLS *in vitro*, suggesting IL-17RA signaling directly contributes to cartilage and bone erosion *via* induction of RANKL. MMPs are considered to participate in the development of arthritis, not only by remodeling the joint structure, but also by proteolytically activating chemokines and cytokines thus amplifying inflammation [42], [43]. Accordingly, we detected enhanced mRNA levels of MMP2, MMP3, and MMP13 in the ankle joints and a considerable decrease in the absence of IL-17RA signaling. In regard to our *in vitro* experiments, MMP3 and MMP13 may also be directly induced by IL-17A in FLS. Although these data suggest activation of these MMPs by IL-17A both *in vivo* and *in vitro*, it is important to note that regulation of MMP activity additionally occurs at the posttranslational level, which may be independent of IL-17RA signaling.

The tumor-like proliferation of FLS and their infiltration into cartilage and bone are a major cause of cartilage and bone erosions in RA. Recently, it has been shown that IL-17RA signaling exerts anti-apoptotic effects *in vivo*, promoting synovial hyperplasia and thus may contribute to the chronicity of RA [44]. We have not addressed possible anti-apoptotic effects of IL-17RA signaling on FLS in our model. The more pronounced difference in cartilage and bone erosions between the wild-type and the *Il17ra^−/−^* group compared to the relatively small difference in clinical signs, particularly in the first 10 days of arthritis, however, suggests that the marked protection of *Il17ra^−/−^* mice from bone and cartilage erosions may be the result of a dual effect of reduced inflammatory mediator production and anti-proliferative effects from lack of IL-17RA signaling.

The K/BxN serum transfer model most closely resembles an acute clinical flare in patients with established RA. Our results suggest that IL-17RA signaling may amplify and sustain arthritis in this clinical situation. Recently, IL-17A has been implicated in sustaining established rheumatoid arthritis [10]. Prior human studies had suggested that the involvement of IL-17A in the pathogenesis of rheumatoid arthritis was limited to its initial phase characterized by the development of an autoimmune response. Our data now lend support to the notion that IL-17RA signaling may play a contributory role in multiple phases of human RA and thus should be considered as a promising pharmacological target for established arthritis. Our data, however, also suggest that inhibition of IL-17RA signaling is unlikely to induce complete remission when used as monotherapy and should be considered as adjunctive therapy.

## Supporting Information

Table S1 Sequences of primers used in this study.
(DOC)
