# Supplementary material for: IL-17RA Signaling Amplifies Antibody-Induced Arthritis
Source: PLoS One. 2011 Oct 20;6(10):e26342. doi: 10.1371/journal.pone.0026342 (PMC3197623; doi:10.1371/journal.pone.0026342)
Supplement: Table S1 — Sequences of primers used in this study. (DOC) [file pone.0026342.s001.doc]

|  | **Forward primer (5' - 3')** | **Reverse primer (5' - 3')** |
| --- | --- | --- |
| **β_2_ microglobulin** | CCGAACATACTGAACTGCTACGTAA | CCCGTTCTTCAGCATTTGGA |
| **IL-1β** | ACCTGTCCTGTGTAATGAAAGACG | TGGGTATTGCTTGGGATCCA |
| **IL-6** | TGTTCTCTGGGAAATCGTGGA | AAGTGCATCATCGTTGTTCATACA |
| **MIP-1α/CCL3** | CCAAGTCTTCTCAGCGCCAT | TCCGGCTGTAGGAGAAGCAG |
| **RANTES/CCL5** | CAAGTGCTCCAATCTTGCAGTC | TTCTCTGGGTTGGCACACAC |
| **MCP-3/CCL7** | TGGGAAGCTGTTATCTTCAAGACA | CTCGACCCACTTCTGATGGG |
| **MIP-1γ/CCL9** | CCCTCTCCTTCCTCATTCTTACA | AGTCTTGAAAGCCCATGTGAAA |
| **KC/CXCL1** | GTGTTGCCCTCAGGGCC | GCCTCGCGACCATTCTTG |
| **MIP-2/CXCL2** | ACGCCCCCAGGACCC | CTTTTTGACCGCCCTTGAGA |
| **LIX/CXCL5** | CTCGCCATTCATGCGGAT | CTTCAGCTAGATGCTGCGGC |
| **IL-17RA** | AGTGTTTCCTCTACCCAGCAC | GAAAACCGCCACCGCTTAC |
| **IL-17RC** | GCTGCCTGATGGTGACAATGT | TGGACGCAGGTACAGTAAGAAG |
| **VEGF-A** | GCTGTGCAGGCTGCTGTAAC | ATGGTGATGTTGCTCTCTGACGT |
| **MIP-3α/CCL20** | TGGGTACTGCTGGCTCACCT | CGAGAGGCAACAGTCGTAGTTG |
| **MIP-3β/CCL19** | ATGCGGAAGACTGCTGCC | CGGAAGGCTTTCACGATGTT |
| **IL-33** | TCCAACTCCAAGATTTCCCCG | CATGCAGTAGACATGGCAGAA |
| **MMP2** | TGATAACCTGGATGCTGTCGTG | CGCTCTTGAGACTTTGGTTCTCC |
| **MMP3** | ACATGGAGACTTTGTCCCTTTTG | TTGGCTGAGTGGTAGAGTCCC |
| **MMP13** | TCCTTGATGCCATTACCAGTC | AAAAAGAGCTCAGCCTCAACCTG |
| **RANKL** | CAGCATCGCTCTGTTCCTGTA | CTGCGTTTTCATGGAGTCTCA |
